# *GNPTAB* c.2404C > T nonsense mutation in a patient with mucolipidosis III alpha/beta: a case report

**DOI:** 10.1186/s12881-018-0679-5

**Published:** 2018-09-12

**Authors:** Chi-Chun Ho, Lilian Li-Yan Tsung, Kam-Tim Liu, Wing-Tat Poon

**Affiliations:** 1 Department of Clinical Pathology, Pamela Youde Nethersole Eastern Hospital, Chai Wan, Hong Kong Special Administrative Region China; 2 Department of Paediatrics & Adolescent Medicine, Pamela Youde Nethersole Eastern Hospital, Chai Wan, Hong Kong Special Administrative Region China

**Keywords:** Mucolipidosis III alpha/beta, Pseudo-hurler polydystrophy, GlcNAc-1-phosphotransferase, *GNPTAB*, Nonsense variant, P.Q802*

## Abstract

**Background:**

Mucolipidosis alpha/beta is an inborn error of metabolism characterized by deficiency of GlcNAc-1-phosphotransferase, in which essential alpha/beta subunits are encoded by the *GNPTAB* gene. The autosomal recessive condition is due to disruptions of hydrolase mannose 6-phosphate marker generation, defective lysosomal targeting and subsequent intracellular accumulation of non-degraded material. Clinical severity depends on residual GlcNAc-1-phosphotransferase activity, which distinguishes between the milder type III disease and the severe, neonatal onset type II disease.

**Case presentation:**

We report the clinical, biochemical and genetic diagnosis of mucolipidosis III alpha/beta in a two-year-old Chinese boy who initially presented with poor weight gain, microcephaly and increased tone. He was confirmed to harbor the common splice site mutation c.2715 + 1G > A and the nonsense variant c.2404C > T (p.Q802*). Clinically, the patient had multiple phenotypic features typical of mucopolysaccharidosis including joint contractures, coarse facial features, kypho-lordosis, pectus carinatum and umbilical hernia. However, the relatively mild developmental delay compared to severe type I and type II mucopolysaccharidosis and the absence of macrocephaly raised the possibility of the less commonly diagnosed mucolipidosis alpha/beta. Critical roles of lysosomal enzyme activity assay, which showed elevated α-iduronidase, iduronate sulfatase, galactose-6-sulphate sulphatase, arylsulfatase B and α-hexosaminidase activities; and genetic study, which confirmed the parental origin of both mutations, were highlighted.

**Conclusions:**

The recently reported nonsense variant c.2404C > T in the *GNPTAB* gene is further recognized and this contributes to the genotype-phenotype spectrum of mucolipidosis alpha/beta.

**Electronic supplementary material:**

The online version of this article (10.1186/s12881-018-0679-5) contains supplementary material, which is available to authorized users.

## Background

Mucolipidosis alpha/beta encompasses a phenotypic spectrum of GlcNAc-1-phosphotransferase (EC 2.7.8.17) deficiency caused by pathogenic variants affecting the alpha/beta subunits-encoding *GNPTAB* gene. Clinical severity and age of onset depend on residual enzyme activity: mucolipidosis type III alpha/beta (MIM 252600) is characterized by significant residual enzyme activity [1], a milder clinical course and a later, childhood, onset compared to the type II (I-cell) disease (MIM 252500), in which minimal enzyme activity causes neonatal disease progressing to childhood death. Despite initial reports of bone marrow transplant successfully delaying cardiopulmonary complications and reversing biochemical or even certain physical abnormalities [2], more extensive case series confirmed the overall poor prognosis and limited reversibility, particularly in the severe form of the disease [3].

At the molecular level, the autosomal recessive disease results from disruptions of the generation of mannose 6-phosphate markers on soluble hydrolases [4], leading to failure of their proper lysosomal targeting and subsequent extracellular loss [5, 6]. These extracellular lysosomal enzymes are nonetheless active and can be detected at elevated levels in the plasma of affected patients [7]. However, due to the missorting of various hydrolases to outside of the lysosomal compartment, there is an accumulation of non-degraded material including oligosaccharides, glycolipids and glycosaminoglycans (GAGs) [8]. These accumulations lead to cellular and organ dysfunction [9, 10], characterized by neuro-developmental, musculoskeletal and cardiopulmonary disorders with lethal complications [7, 11].

In this study, we report a two-year-old boy being diagnosed of mucolipidosis III alpha/beta, with initial presentation of poor weight gain, microcephaly and increased tone at five months of age. Subsequent follow up showed evolving coarse facial features, musculoskeletal abnormalities including craniosynostosis, joint contractures and developmental delay which prompted a series of biochemical investigations. A dried blood spot (DBS) enzyme activity assay found increased activity of multiple lysosomal enzymes, which pointed to mucolipidosis II/III. Targeted genetic study of the *GNPTAB* gene was performed on the index patient and his parents, confirming the patient as a compound heterozygote with c.2715 + 1G > A and the c.2404C > T (p.Q802*) nonsense mutation, reported shortly after the initial submission of this article [12]. In light of the genetic findings, the clinical history of the patient is reviewed, and the management and future treatment options for this patient are discussed.

## Case presentation

### Case description and clinical examination

**Fig. 1:**
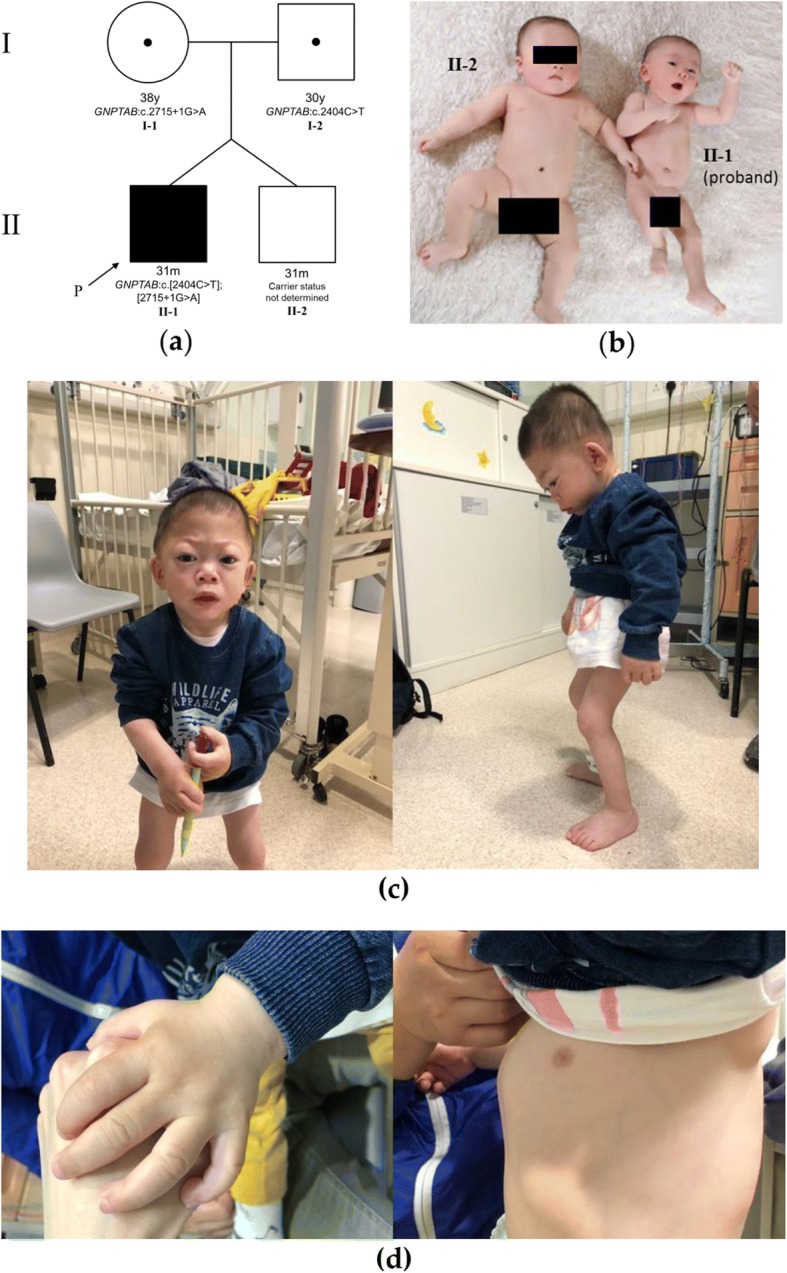
Clinical progression of the mucolipidosis III alpha/beta phenotype. **a** Pedigree of the family. Both parents were confirmed to be heterozygous carriers of the respective pathogenic variant. **b** The twin brother (II-2) and the proband (II-1) at four months of age. The current photo shows a clear lack of catch-up growth even at four months of age for the proband Note that facial dysmorphism was minimal. **c** At 24 months of age, the coarsening of facial features, trigonocephaly, flat nasal bridge and prominent eyes became more apparent (left). The kypho-lordosis, crouched gait with bent knee posture was seen on standing. Claw-hand deformities were seen (right) (**d**) Close-up view of the thick claw hand of the patient (left) and pectus carinatum deformity of the chest wall (right)

The proband was born of a non-consaguineous, in-vitro fertilization dichorionic twin pregnancy at 36 weeks of gestation. The father and mother are Han Chinese, aged 30 and 38 years respectively (Fig. 1a). Except for previously treated maternal Graves’ disease, there was no significant family history including neurodevelopmental delay or metabolic disease. While the twin brother had a birth weight of 2.50 kg, the proband was small for gestational age with birth weight 1.79 kg, with length and head circumference all below the 3rd percentile (Fig. 1b). Antenatal and perinatal history was unremarkable. The proband was discharged on day 16 with body weight of 2.16 kg.

He was first referred to the authors’ Paediatric out-patient clinic for poor weight gain, microcephaly and increased tone over all four limbs at five months of age (corrected age of four months). Head circumference and weight were below the third centile. When reviewed at seven months old, physical examination showed failure to thrive with body weight and height less than the third centile, head circumference at the third centile, plagiocephaly, and a closed anterior fontanelle. There were no obvious dysmorphism or neurocutaneous stigmata. Chest, cardiovascular and abdominal examination was normal with no organomegaly. There was a reducible left inguinal hernia and the genitalia were normal. However, flexion contractures affecting both knees and tight hip adductors were noted. There was also increase in tone over the lower limbs with brisk deep tendon reflexes. Developmental assessment showed mild gross motor delay.

### Imaging and laboratory investigations

In view of the abnormal physical findings at the age of seven months, investigations include complete blood count and routine biochemical analysis of liver and renal function were done. The results were all within age-specific reference intervals. Blood lactate, pyruvate and glucose levels were normal. Thyroid stimulating hormone was not elevated. Urine cytomegalovirus culture was negative. Urine metabolic screening for ketones, reducing sugars, amino acids, phenylpyruvic acid, tyrosine metabolites, cysteine & homocysteine and keto acids were negative. DBS metabolic screening found unremarkable patterns of amino acids and acylcarnitines. Skull X-ray and non-contrast magnetic resonance imaging (MRI) of the brain did not reveal frank radiographic evidence of craniosynostosis or definite abnormal signal intensity in the brain.

**Fig. 2:**
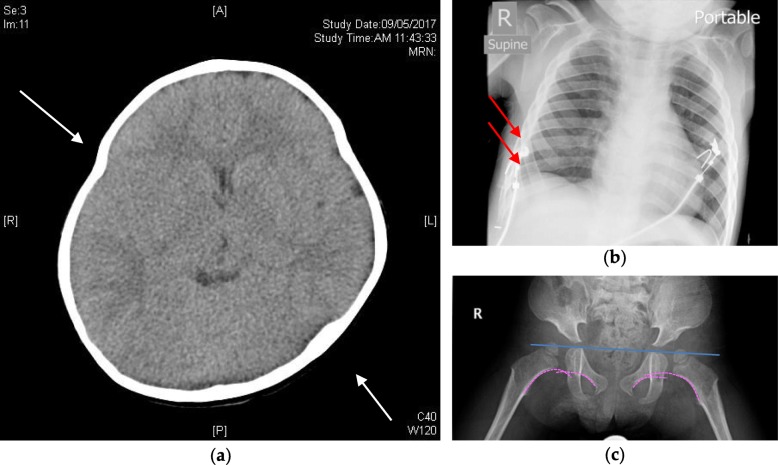
Imaging investigations of skeletal deformities. **a** Non-contrast computed tomography scan of the brain at 21 months, showing the deformed skull shape (white arrows). The scan also revealed other abnormalities as described in-text. **b** Subtle widening of the ribs was noted (red arrows) on retrospective review of a chest X-ray taken on an admission for viral infection at 28 months. **c** Hip X-ray at 30 months, showing subluxation of bilateral hip joints. The femoral heads are above the Hilgenreiner’s line (in blue) and bilateral Shenton lines (dashed, in pink) show discontinuation

Follow up imaging with X-ray and MRI spine at 13 months old showed anterior subluxation of the cervical spine at C1/2, posterior hemivertebra at L2 and a mild kyphosis. Computed tomography (CT) of the brain at 14 months old showed fusion of metopic suture and narrowing of sagittal suture. A repeated scan at 21 months old showed craniosynostosis with slightly deformed skull shape, partial fusion of coronal suture near the vertex, sagittal suture and bilateral lambdoid sutures (Fig. 2a). Retrospective review of a previous chest X-ray film found mildly widened ribs and scalloped vertebrae (Fig. 2b). With clinical evolving kypho-lordosis and knee and hip flexion contractures and radiological evidence of bilateral hip subluxation (Fig. 2c) a syndromal diagnosis was suspected.

Subsequent regular follow up by the neurologists at 26 months of age showed persistent failure to thrive, with progressive evolvement of coarse facial features (including prominent eyes, microcephaly with craniosynostosis, low set ears, thick lips, thick spade-shaped claw hands), musculoskeletal abnormalities (kypho-lordosis, contracture over hip, knee, elbow and wrist and obvious pectus carinatum) (Fig. 1c) and mild global developmental delay involving the gross motor, fine motor and verbal aspect. They together raised the suspicion of mucopolysaccharidosis (MPS) or an MPS-related condition. Of note, there was no hepatosplenomegaly, and the child was noted to have microcephaly. Spot urine testing was performed and found an increased mucopolysaccharide excretion (acid mucopolysaccharide to creatinine ratio of 25.9 mg/mmol, normal: < 15 mg/mmol). No pathological pattern was detected on mucopolysaccharide electrophoresis or oligosaccharide thin-layer chromatography.

Because of the equivocal findings, the DBS and urine samples of the proband were sent to a reference laboratory (Laboratory of Biochemical Genetics, Department of Medical Genetics, National Taiwan University Hospital) for lysosomal enzyme activity and quantitative urine GAGs testing. DBS enzyme activity testing showed increased activity of α-iduronidase (23.96 μM/h, normal: > 1.32 μM/h), iduronate sulfatase (210.78 μM/h, normal: > 4.45 μM/h), galactose-6-sulphate sulphatase (6.26 μM/h, normal: > 1.02 μM/h), arylsulfatase B (38.77 μM/h, normal: > 3.45 μM/h) and α-hexosaminidase (76.35 μM/h, normal: > 1.61 μM/h). Urinary GAG excretion was normal. While no upper normal limits were cited for the lysosomal enzyme activities by the testing laboratory, their generalized elevation in the clinical context was suggestive of mucolipidosis [13].

### Genetic testing and Cascade screening

Sanger sequencing analysis was performed for all coding exons and respective 10-basepair flanking regions of the *GNPTAB* gene. PCR primers were designed using an in-house developed software (AutoPrimer, version 1.0) with automatic splitting/joining of adjacent exons, avoidance of primer-binding site nucleotide polymorphisms and systematic checking for potentially non-specific amplifications (Additional file 1: experimentally validated PCR and sequencing primers for *GNPTAB* gene). Genomic DNA extraction, target amplification and DNA sequencing were performed as previously described [14]. Briefly, DNA from peripheral blood was extracted using Qiagen QIAamp® DNA Blood Mini Kit (Qiagen, Hilden, Germany) following the manufacturer’s instructions. Target exons were amplified from extracted genomic DNA by PCR; each 25 μL reaction contains: 12.5 μL AmpliTaq Gold® 360 Master Mix (Applied Biosystems, CA, USA), 1.0 μL 360 GC Enhancer, 25 μM of each of forward and reverse primers, 20 ng purified genomic DNA and 7.5 μL of PCR-grade water. Amplification was performed using a standardized stepdown PCR protocol. Sanger sequencing was performed using the BigDye Terminator v1.1 Cycle Sequencing Kit (Applied Biosystems, CA, USA) and an ABI 3500 genetic analyzer [15].

Heterozygous *GNPTAB* (NM_024312.4) c.2404C > T (p.Q802*) and c.2715 + 1G > A variants were detected in the proband. The c.2715 + 1G > A variant has been previously reported in compound heterozygosity with other nonsense mutations [16] and the c.2404C > T (p.Q802*) nonsense variant was reported by Wang et al. [12] shortly after the initial submission of the present article in May 2018. The recently reported variant c.2404C > T was absent from normal controls in the 1000 genome [17] and ExAC database [18]. To confirm compound heterozygosity in the proband, PCR and Sanger sequencing for the respective locations were performed for both parents using the same primers and identical conditions as described. The c.2404C > T variant was detected in heterozygous state in the father and the c.2715 + 1G > A variant was detected in heterozygous state in the mother; both parents were negative for the other variant, compatible with their asymptomatic phenotype (Fig. 1a). These together classify the recently reported variant as being pathogenic, according to the American College of Medical Genetics 2015 Guidelines [19]. Carrier testing for the asymptomatic twin brother of the proband was declined by the parents.

## Discussion and conclusions

In this study, we reported a two-year-old boy confirmed to have mucolipidosis III alpha/beta due to compound heterozygous pathogenic variants in the *GNPTAB* gene. Cascade screening revealed the parental origin of both mutations, including the common splice site variant c.2715 + 1G > A, accounting for up to 28% of pathogenic variants in a Chinese cohort [20], and the recently reported nonsense variant c.2404C > T (p.Q802*). Clinically, the patient had multiple phenotypic features of mucolipidosis III alpha/beta which mimicked manifestations of MPS, including gradual evolvement of large and small joint contractures, coarse facial features, kypho-lordosis with pectus carinatum, umbilical hernia and developmental delay [21]. A differentiating clinical feature in this case, however, was the presence of microcephaly - which would be atypical of MPS – despite being a common feature of mucolipidosis alpha/beta and even the main presenting sign in some patients [22, 23]. Developmentally, the degree of delay in this patient was relatively mild compared to more commonly seen MPS cases in the region [24], particularly type I and type II MPS in which patients can manifest severe complications early [25]. These clinical features, while not absolute, should nevertheless alert the physician the possibility of mucolipidosis in patients being investigated for suspected MPS. Retrospectively, the subtle dysmorphic features could have been noticed earlier but these insidious clinical features were also partly masked by the baby’s medical history of prematurity. Clinicians’ awareness of the disorder could have been improved, despite the rarity of the disorder. Also, universal newborn screening for lysosomal storage disorders could have helped in early diagnosis [26], although such testing is currently not available in Hong Kong.

The generalized increase in plasma lysosomal enzyme activity provided an important diagnostic clue to establishing the biochemical and genetic diagnosis in this case. While specific patterns of enzyme activity elevation has been reported and applied in simplified, targeted screening strategies [27], it should perhaps be emphasized that only the use of multi-enzyme activity paneling would allow the most sensitive detection simultaneous differentiation from the phenotypically-similar MPSs and other inborn metabolic defects [28]. Of note, the more recently developed tandem mass spectrometry assays have allowed the determination of multiple lysosomal enzyme activity [29, 30] and GAG levels [31] from a DBS sample, which can be transported to a reference laboratory by regular mail. With the increasing inclusion of lysosomal storage diseases in newborn screening programmes [26, 32], it is anticipated that the pre-symptomatic detection, particularly of the childhood-onset mucolipidosis and MPS, will allow proactive management and better prevention of complications, even if specific or curative treatments may not be available.

Unlike MPS, in which enzyme replacement therapies have been made available or undergoing clinical trials for multiple subtypes [33–36], and bone marrow transplant has shown various degrees of success [37], current treatment options of mucolipidosis alpha/beta remain limited. Management for patients with mucolipidosis alpha/beta, particularly type III (attenuated type) patients who may survive into their thirties [38], still focused on the conservative management of the skeletal, cardiac and pulmonary complications. Understandably, due to the pervasive effect on lysosomal enzyme targeting of the GlcNAc-1-phosphotransferase defect, no single lysosomal hydrolase replacement would be effective in treating the condition. Replacement of the GlcNAc-1-phosphotransferase by gene therapy has yet to make its way into human trials [39]. More recent reports and cohorts on allogeneic hematopoietic stem cell transplantation (HSCT) [3, 40, 41] rarely reproduced the marked clinical improvement in early reports [2, 42]. In the USA cohort, it was found that post-transplant prognosis significantly depends on pre-existing disease status [3]. A major limitation, as in the current study, was that most of these HSCT cases were diagnosed only by lysosomal enzyme activity assay and confirmed by gene sequencing; the severity of pathogenic variants were not confirmed at the expression and protein levels. As actual disease severity correlates with residual GlcNAc-1-phosphotransferase activity and resultant lysosomal function, rather than the variably elevated levels of extracellular enzyme activity (representing a combined effect of mis-targeting and upregulation) it remains unclear whether the HSCT-responsive cases could actually be due to residual GlcNAc-1-phosphotransferase activity compatible with a milder phenotype – and thus other mucolipidosis type III patients, as in our case, may benefit from such.

At the age of 23 months, the patient was formally assessed by the Child Assessment Centre (Department of Health, Hong Kong Special Administrative Region, China) and was commented as having mild global delay. Re-assessment by the authors at age of 34 months showed a gross motor and fine motor development corresponding to normal children of 20–22 months, a cognitive function (non-verbal aspects) corresponding to normal children of 18–20 months and a verbal ability corresponding to Chinese children of about 2.5 years of age (able to speak 3–4 word phrases). The patient’s vision and hearing were normal.

## Additional file

Additional file 1: Primers used for PCR and sequencing of *GNPTAB* gene. Eighteen pairs primers were designed with the help of in-house software AutoPrimer 1.0 (https://github.com/autoprimer/1.0 or https://github.com/hkhcc/seq_processing for the latest developmental version). The primer sequences and the corresponding *GNPTAB* exons corresponding to the transcript NM_024312.4 are listed. (DOCX 21 kb)

## Abbreviations

CT: Computed tomography
DBS: Dried blood spot
GAG, GAGs: Glycosaminoglycan, glycosaminoglycans
GlcNAc-1-phosphotransferase: N-acetylglucosamine-1-phosphotransferase
HSCT: Hematopoietic stem cell transplantation
MIM: Mendelian Inheritance in Man database
MPS: Mucopolysaccharidosis
MRI: Magnetic resonance imaging
